# Kounis Syndrome: Allergic Vasospastic Cardiac Event

**DOI:** 10.7759/cureus.10498

**Published:** 2020-09-16

**Authors:** Shima Sidahmed, Saadia Shafi, Areeg Bala, Anwar Zaitoun, Ghassan Bachuwa

**Affiliations:** 1 Internal Medicine, Michigan State University/Hurley Medical Center, Flint, USA; 2 Cardiology, Covenant HealthCare, Saginaw, USA

**Keywords:** anginal chest pain, drug allergy recording, left heart cath, cad

## Abstract

Kounis syndrome (KS) is an acute coronary event secondary to an allergic reaction. It is provoked by environmental agents, food, and medications. KS is caused by the release of allergic mediators. We are reporting a case of a 39-year-old man who had a syncopal episode after he took cephalexin and ibuprofen for toothache. He developed chest pain and erythematous rash later. His electrocardiography did not show any ST-segment elevation changes and cardiac troponins were elevated. He was started on the acute coronary syndrome treatment protocol. Coronary angiography revealed no significant obstructive or culprit lesions. The patient was discharged home in stable condition. He is advised to adhere to lifestyle modification and outpatient follow-up with cardiology and allergy/immunology. KS is infrequently reported in the medical literature. Physicians should pay attention to any allergic reaction preceding the acute cardiac event and consider KS in the differential diagnosis.

## Introduction

Kounis syndrome (KS) is an acute myocardial infarction (MI) triggered by allergic reaction or anaphylaxis provoked by medications, food, or environmental triggers. The most common causes are antibiotics and insect's bites [1]. KS is attributed to the degranulation of mast cells and the release of inflammatory and allergic mediators, including histamine, neutral proteases, and arachidonic acid products [2]. KS is classified into three types: coronary vasospasm with normal coronary arteries, acute MI with underlying coronary artery disease (CAD), or coronary thrombosis. It was first recognized by Kounis and Zavras in 1991 as "allergic angina syndrome'' [1]. The incidence of KS is not reported due to the paucity of cases as well as underdiagnosis of this pathology. Here we present a patient without known allergies, who presented to the emergency department (ED) with syncope and chest pain, in the setting of acute allergic drug reaction.

## Case presentation

A 39-year-old Caucasian male presented with a known medical history of type 2 diabetes mellitus, hypothyroidism, obesity, dyslipidemia, and tobacco smoking. He recently had a toothache and dental infection. The patient was evaluated in the ED with syncope, chest pain, and symptoms suggestive of a possible allergic reaction after taking the first doses of cephalexin and ibuprofen. The patient was brought to the ED by his family after a witnessed syncopal episode at home. According to his family, three minutes after taking the medications, the patient vomited, and started having numbness in his lips and arms along with blurry vision and lightheadedness. The patient then fainted for seconds and regained consciousness in less than a minute.

En route to the hospital, he reported a chest pain that was associated with profuse sweating. He was given sublingual nitroglycerin, aspirin, and morphine. The chest pain resolved shortly. Upon initial assessment in the emergency room (ER), the patient had an episode of hypoxia with oxygen saturation (SpO_2_) of 80%, and hypotension with a blood pressure of 90/60 mmHg. He became lethargic and confused and developed a generalized itchy skin rash. He received intramuscular injection of epinephrine with the concern of allergic reactions.

**Figure 1:**
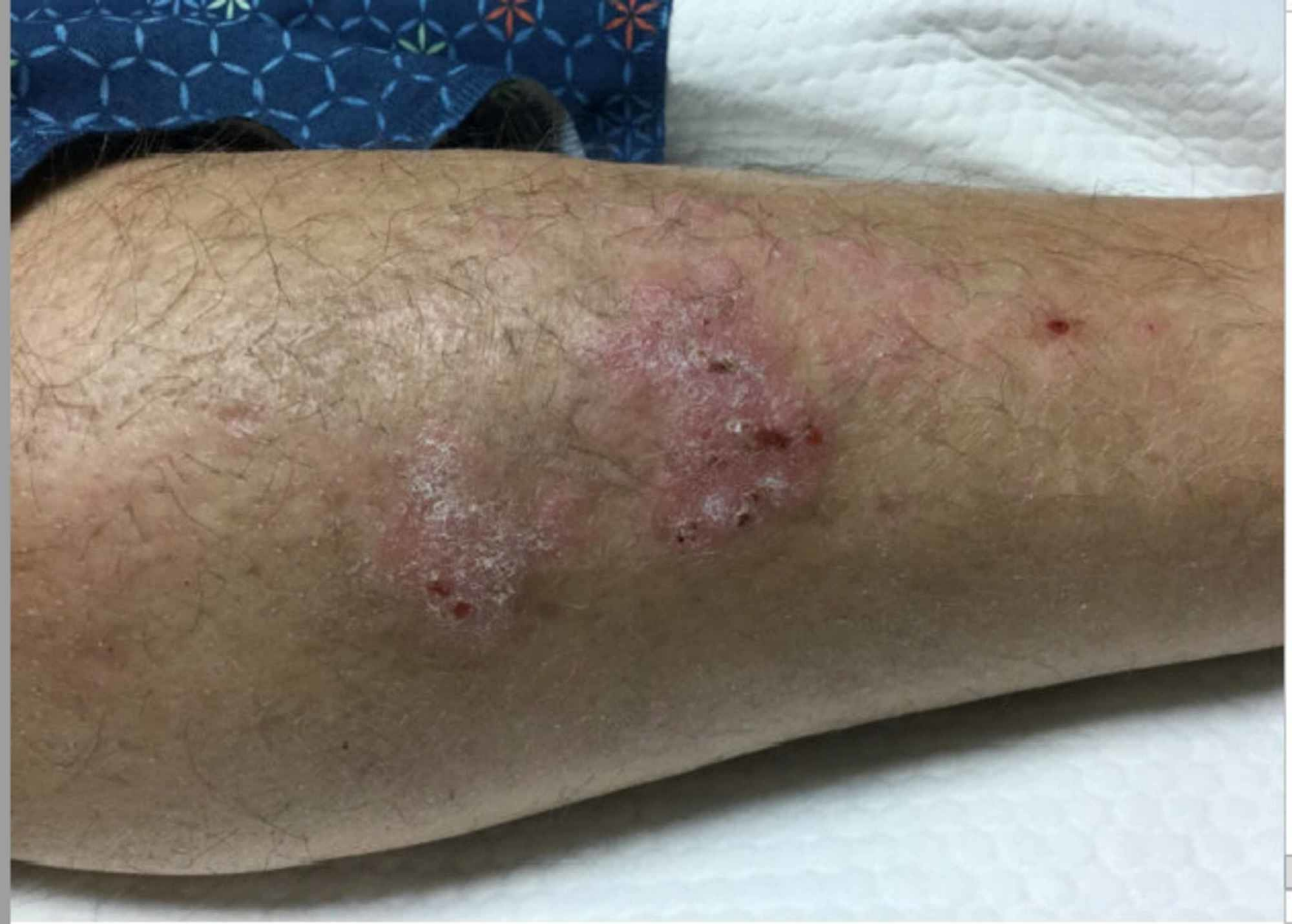
Erythematous skin rash on patient’s left upper extremity

On examination, the patient was alert and oriented to time, place, and person. His vital signs stabilized. The cardiopulmonary exam revealed no abnormalities. A diffuse erythematous skin rash was noted on patient's upper extremities (Figure 1).

**Figure 2:**
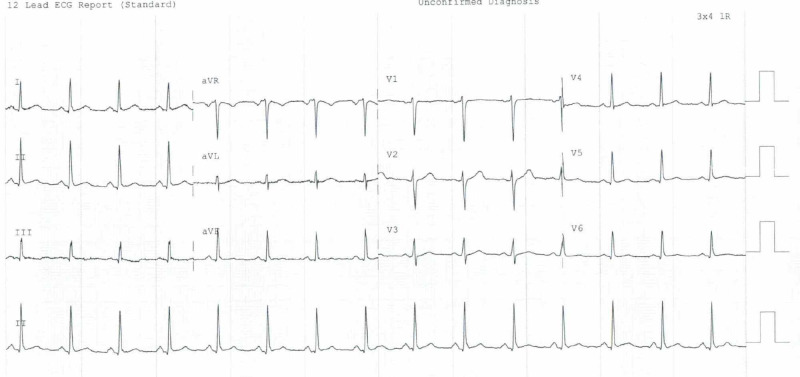
Electrocardiogram (EKG) illustrates normal sinus rhythm without acute ST-segment elevation

Electrocardiography (EKG) showed normal sinus rhythm, without significant ST elevation (Figure 2). Labs showed leukocytosis of 18,200/µL (4,000-10,800/µL) with normal hemoglobin and platelets count on a complete blood count (CBC). The coagulation panel was unremarkable. Cardiac troponin came back elevated at 2.62 (0.00-0.04 ng/ml), trended up subsequently to 9.08 ng/ml and peaked at 15.59 ng/ml, and then decreased within 24 hours from initial presentation. 

**Figure 3:**
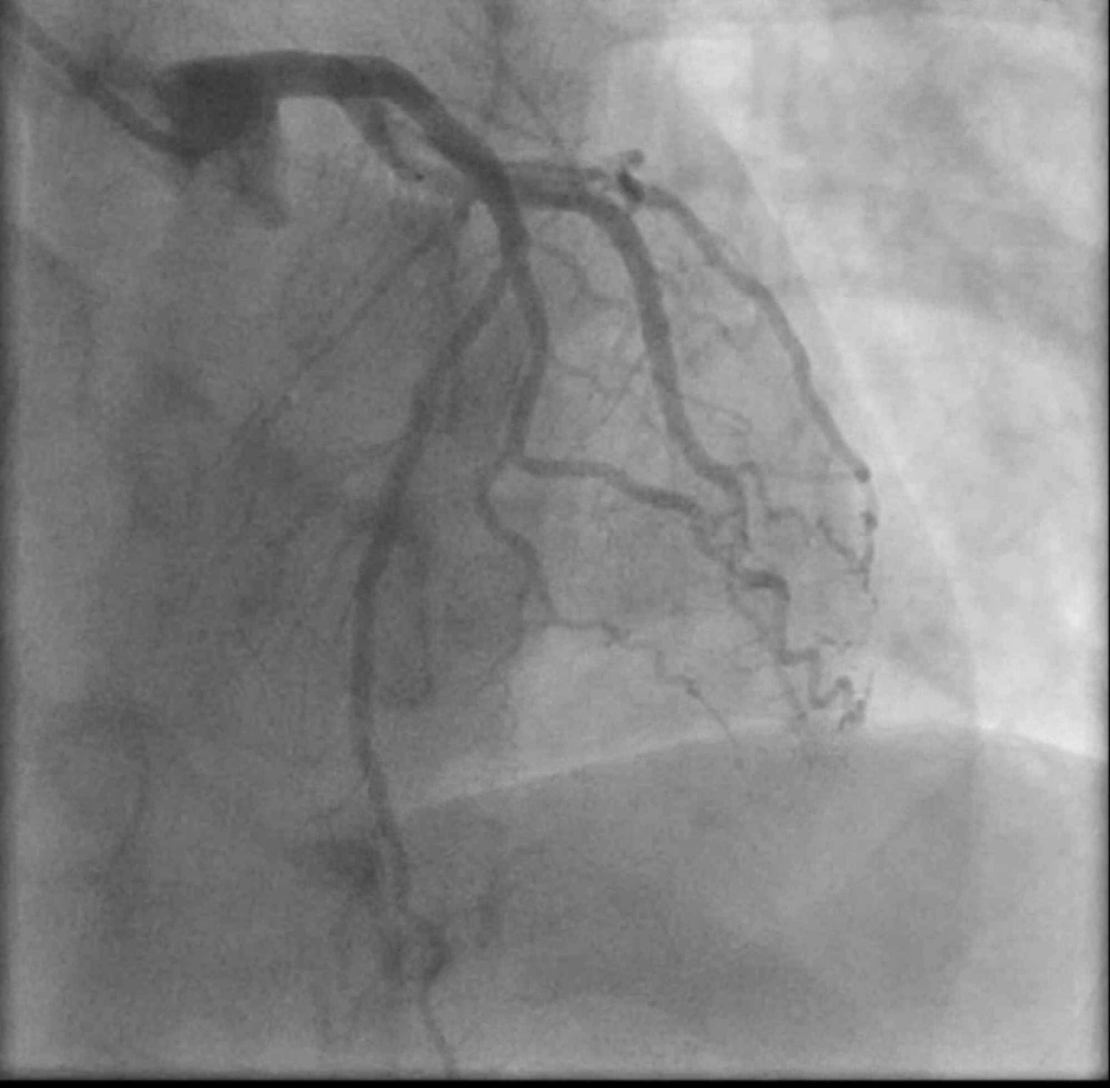
Coronary angiography showing normal coronary circulation

The patient was started on an acute coronary syndrome protocol for non-ST elevation myocardial infarction (NSTEMI). He underwent cardiac catheterization, and his coronary angiography did not show any evidence of coronary artery disease or obstructive lesions (Figure 3).

No intervention was performed. His echocardiogram revealed normal left ventricular function without evidence of structural heart disease. The patient's clinical presentation was believed to be due to coronary vasospasm in a setting of allergic reaction consistent with KS type 1. The patient was treated medically with aspirin, and high-intensity statin for primary prevention of coronary artery disease. He was also advised and counseled about lifestyle modification in addition to avoidance of cephalosporins. 

## Discussion

KS is an underrecognized cause of allergic vasospastic acute coronary events. According to a recent review article [2] published in 2017 involving 175 cases of KS, it is common among the age group between 40 and 70 years. Patients were noted to have comorbidities, including hypertension, diabetes mellitus, dyslipidemia, and smoking.

The pathophysiology of KS entails allergic reactions causing the release of inflammatory mediators, including histamine, neutral proteases, arachidonic acid products, platelet-activating factor, cytokines, and chemokines [3]. The concept of mast cells causing damage to the coronary artery was pathologically established when the accumulation of mast cells (200-fold more) was found at the site of atheromatous erosion or rupture in 20 patients who died of acute myocardial infarction in a study published in 1995 [4].

As we mentioned earlier, it has three subclasses and type 1 was the most common (72.6%) followed by type 2 (22.3%) and type 3 (5.1%) [2]. In type 1 KS, the inflammatory mediators will induce coronary vasospasm in the setting of normal vasculature with or without troponin leak. In type 2, plaque erosion or rupture will accompany the coronary spasm leading to acute myocardial injury. Type 3 group is mainly due to thrombosis secondary to the allergic response and subsequent platelet activation.

Our patient's clinical presentation is likely due to the KS type 1 variant as he has no evidence of obstructive lesions on cardiac catheterization. Key factors that led to identifying this patient to have KS included exposure to potential allergen followed by acute anginal episode and erythematous skin rash. Previously, case reports have found an association with cephalosporins, including cefazolin [5], as well as fluoroquinolones, including ciprofloxacin [6]. Furthermore, KS incited by ibuprofen has been reported previously causing type 1 variant [7]. Our patient took oral cephalexin and ibuprofen concurrently, and hence it is difficult to identify a sole culprit drug or combined effect. The patient reports no previous allergy to either medication.

The acute management of KS relies on subtype. The most used medications in type 1 are steroids and antihistamines for mast cell stabilization to control the allergic reaction and end the response cascade [7]. Fluid resuscitation is also necessary to adequately support blood pressure. Morphine should be avoided in suspected patients as it can increase histamine release resulting in worsening coronary vasospasm. In the acute setting, beta-blocker use can cause unopposed alpha-adrenergic activity, further reducing coronary perfusion. Epinephrine is commonly used in an anaphylactic reaction; however, in KS patients close cardiac monitoring is needed as epinephrine can induce coronary vasoconstriction as well [8].

In the other subgroups (2 and 3), treatment of the coronary event should be similar to other causes, including both medical and interventional management according to the most recent acute coronary syndrome (ACS) guidelines [2]. The use of nitrates can be considered given its known coronary vasodilatation effect. However, it can result in a further drop in blood pressure [9]. Among all subgroups, avoidance of the causative agent is crucial and the mainstay treatment.

KS has a good outcome, and it is associated with rapid recovery and fewer complications. Serious complications happened only in a small percentage of the patients as follows: cardiogenic shock (2.3%), cardiac arrest (6.3%), and cardiac death (1.1%) [2].

## Conclusions

Failure to recognize KS contributes to its underdiagnosis. Physicians should pay close attention to signs and symptoms of allergic reaction preceding the acute cardiac event and should consider KS in the differential diagnosis. KS has overall good prognosis and outcome; therefore, a multidisciplinary team from cardiology and allergy/immunology is recommended for the management of KS and post-hospital discharge follow-up.
